# The effect of teacher support on Chinese university students’ sustainable online learning engagement and online academic persistence in the post-epidemic era

**DOI:** 10.3389/fpsyg.2023.1076552

**Published:** 2023-01-30

**Authors:** Xinglong Ma, Man Jiang, Liying Nong

**Affiliations:** ^1^Chinese International College, Dhurakij Pundit University, Bangkok, Thailand; ^2^Department of Basic Science, Guizhou Aerospace Vocational and Technical College, Zunyi, China; ^3^School of Education and Music, Hezhou University, Hezhou, China

**Keywords:** post-epidemic era, Chinese university students, perceived teacher support, sustainable online learning engagement, online academic persistence

## Abstract

Since entering the post-epidemic era of COVID-19 at the end of 2021, schools have mostly adopted a combined online and offline teaching mode to effectively respond to the normalized epidemic, which has changed the traditional learning mode of students. Based on the study demand-resources (SD-R) model theory, this study developed a research model and proposed six research hypotheses to explore the relationship between Chinese university students’ perceived teacher support (PTS), online academic self-efficacy (OAS-E), online academic emotions (OAE), sustainable online learning engagement (SOLE), and online academic persistence (OAP) in the post-epidemic era. In this study, 593 Chinese university students were invited to respond to a questionnaire survey using the convenience sampling method. The results of the study showed that: PTS had a positive effect on OAS-E and OAE; OAS-E had a positive effect on OAE; OAS-E and OAE had a positive effect on the students’ SOLE; and SOLE had a positive effect on their OAP. Based on the analysis, it is recommended that teachers provide more support and resources to further enhance students’ academic self-efficacy and academic emotions, and thus ensure students’ SOLE and OAP.

## 1. Introduction

To eradicate poverty, implement quality education, and achieve human well-being and development, in 2015, the 17 Sustainable Development Goals (SDGs) were established by the United Nations (UN), which also provided a new vision and goals for action for sustainable development (United Nations, 2015). Among them, Sustainable Development Goal 4 (UN’s SDGs4) purposes at how to achieve quality education and promote lifelong learning opportunities for all to better achieve sustainable development in education (UN, 2020). However, the persistence of the COVID-19 epidemic has affected the implementation of the SDG process. Moreover, since the end of 2021 in the post-COVID-19 era, the associated risk and regular epidemic prevention has had a significant influence on individual’s social and economic livelihoods (Cheng et al., 2021), including in the field of education. As the COVID-19 epidemic continues to recur, many schools are rapidly adopting online delivery models so that students can continue their education online (Wang et al., 2021), which not only changes the traditional learning patterns of students, but also makes the continued implementation of online learning an important issue for the sustainability of education in the post-epidemic era.

In order to effectively respond to the complex situation of the epidemic, in February 2020, the Chinese Ministry of Education issued the Guidance on the Organization and Management of Online Teaching and Learning in General Universities during Epidemic Prevention and Control. Many Chinese universities have adopted online or non-face-to-face learning approaches to transform traditional educational activities into digital teaching and learning modes (Putra et al., 2019; Ye et al., 2022). Online learning plays a crucial role in the current educational environment because it can solve many of the problems of traditional offline teaching and learning. The present online teaching cases have more flexibility (Fagö-Olsen et al., 2020), and more importantly, online learning helps students to learn online anytime across geographical space (Maatuk et al., 2022). However, the sustainable online learning engagement (SOLE) of university students in China during the epidemic blockade is not optimistic, as evidenced by the low motivation of students to engage in online learning (Yang et al., 2021) and their tendency to be distracted from completing online learning tasks in a focused and sustained manner, which may even lead to university students in China dropping out or losing their enthusiasm for learning (Lee et al., 2019). Whereas previous studies have found that students’ academic persistence as an important variable in online learning environments, their academic persistence not only affects their willingness and behavior to engage in online learning, but may also influence the effectiveness of their online learning (Yu et al., 2022). More importantly, higher academic persistence of students will lead to higher online learning engagement status (Jung and Lee, 2018). Therefore, in the context of the post-epidemic era, how to promote SOLE and online academic persistence (OAP) among university students has become an essential matter for the sustainable development of education in the post-epidemic era.

According to SDG4, the educational environment and student behavioral outcomes are interconnected and act in a sustainable system that continuously and dynamically influences student behavior and outcomes (Tran et al., 2020). That is, the recurrence of COVID-19 has brought about changes not only in the learning environment but also in the learning patterns of students (Hong et al., 2021a,b). In addition, considering that teacher support is an important social resource, during the COVID-19 pandemic, teacher behavior not only affects students’ motivation to learn online but also affects their level of engagement in online learning (Adeshola and Agoyi, 2022). Related studies have found that when conducting online courses such as Small Private Online Courses (SPOCs) and Massive Open Online Courses (MOOCs), the lack of effective communication and interaction between teachers and students and the failure to understand students’ learning dynamics and participation status in a timely manner have led to a low level of student participation in such courses (Liu et al., 2019; Shen and An, 2022). In addition, prior to the outbreak, due to the lack of face-to-face instruction and the large spatial distance between learners and instructors, many students tend to be less engaged in online learning than in traditional learning environments, which has a more significant impact on their sustainable and effective engagement in online learning (Kim et al., 2019). While the implementation of online learning has become an essential and regular mode of instruction in the post-epidemic era, it is not yet known how faculty support will influence student engagement and OAP in online learning.

Furthermore, the study demand-resources (SD-R) model is based on the JD-R model theory and focuses on the relationship between student learning requirements, learning resources and health. According to the SD-R model recommended by Lesener et al. (2020), study resources can facilitate student engagement and produce positive learning outcomes in the larger educational context. In other words, when taking online courses, students interact with study resources in a two-way manner and are connected to the educational environment and system in a continuous and dynamic way (Koob et al., 2021). Study resources can therefore influence not only students’ motivation but also their learning behaviors and outcomes (Robins et al., 2015). Researchers have also explored the relationship between learning engagement and psychological well-being among university students based on the SD-R model and found that more study resources can influence students’ individual resources, which in turn affects aspects of their psychological well-being (Wei et al., 2022). Related research has found that students may be more actively engaged in course learning with teacher support and engage in learning activities with a higher sense of academic self-efficacy (Lauermann and ten Hagen, 2021). At the same time, if students can feel happy and relaxed in online courses, it will not only facilitate their active participation in online learning activities, but also influence their performance in a sustainable and positive way (Jdaitawi, 2020). As the SD-R model theory suggests, more environmental and individual resources are closely related to student behavior and outcomes (Lesener et al., 2020). That is, environmental resources such as teacher support may influence students’ academic performance and persistence through their academic self-efficacy and academic emotions.

However, most studies on online learning during COVID-19 explored the relationship between students’ academic performance and mental health or focused on students’ learning satisfaction in different online courses (Yekefallah et al., 2021). Fewer studies have discussed the aspects of supporting university students’ engagement and intention to learn through environmental resources such as teachers (Jin et al., 2021), or of further promoting university students’ engagement and persistence by stimulating their OAS-E and sustaining their positive academic emotions. However, in the current post-epidemic context, it has become important to explore how faculty can support university students’ online learning engagement. Thus, this study explored the association among perceived teacher support (PTS), online academic self-efficacy (OAS-E), online academic emotions (OAE), SOLE, and OAP in online course learning in a post-epidemic context based on the SD-R model theoretical framework and SDG4.

## 2. Research model and hypothesis

In this chapter, this study constructed the framework diagram of this study model based on the existing literature and theoretical models, and proposes the hypothetical relationships among the research variables in conjunction with the theoretical models and relevant literature exploration.

### 2.1. Research model

**Figure 1:**
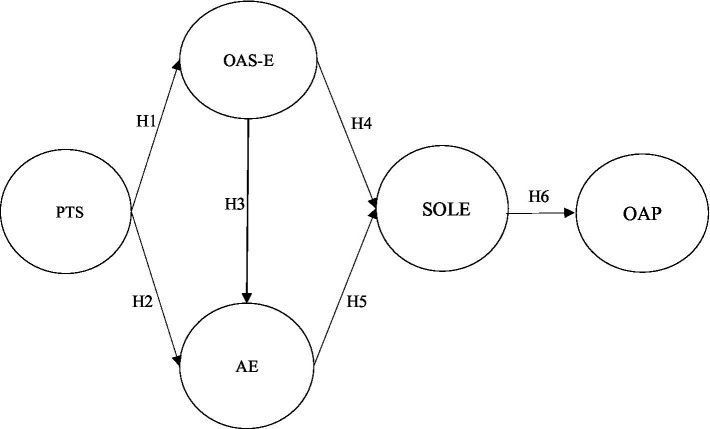
Research model. Perceived teacher support (PTS), online academic self-efficacy (OAS-E), online academic emotions (OAE), sustainable online learning engagement (SOLE), and online academic persistence (OAP).

According to the SD-R model, study resources support student engagement and positive study outcomes (Lesener et al., 2020). Study resources include environmental resources (e.g., perceived teacher support, classroom climate, etc.), personal resources (e.g., academic self-efficacy and psychological resilience), and learning behaviors including learning engagement (Wei et al., 2022). Thus, PTS can be considered as an environmental resource, OAS-E and academic emotions can be considered as personal resources, and SOLE and OAP can be considered as learning outcomes and behaviors. That is, based on the post-epidemic context, the higher the PTS among university students in the current online learning environment, the more likely it is to be conducive to motivating their OAS-E and academic emotions, which will continue to influence their online learning engagement and OAP. Therefore, this study investigated the relationship between university students’ PTS, OAS-E, OAE, SOLE, and OAP by combining the SD-R model theory and mapped the research model structure as follows (Figure 1).

### 2.2. Research hypothesis

#### 2.2.1. The effect of perceived teacher support on online academic self-efficacy and online academic emotions

In SDG4, teachers are an important factor influencing educational sustainability, because teachers are not only the primary guides of their students, but they also continue to influence student behavior and development (Bengtsson et al., 2020), and in related research it has been found that more teacher support not only motivates students to engage in course learning, but also motivates student self-efficacy, which in turn impacts their academic achievement and performance (Lauermann and ten Hagen, 2021). Moreover, self-efficacy is seen as a key factor in motivating learners in online courses. It is the confidence and beliefs that learners have about their own task completion (Bandura, 1977; Berga et al., 2021), and it influences learners’ behavioral outcomes in specific contexts. With the COVID-19 pandemic, instructors have become increasingly important for student learning in online courses such as MOOCs and SPOCs. Those with more support tend to have higher academic self-efficacy and the support continues to influence student engagement in online learning (Alamri, 2022). In addition, due to the impact of the epidemic, many schools have adopted online teaching models or non-face-to-face approaches, which are prone to a lack of effective and sustainable positive teacher-student interactions which then affect the students’ OAS-E (Gao et al., 2021; Adeshola and Agoyi, 2022). That is, in the post-epidemic context, more PTS would be beneficial for stimulating students’ OAS-E. Grounded on the above literature, this study proposed the following research hypothesis:

*H1*: PTS has a positive effect on OAS-E.

According to the SD-R model theory, environmental resources such as social, teacher, and family support can better support students’ development (Wei et al., 2022), while teacher support, as an important social resource, plays a crucial role in promoting student behavior and development and can be more involved in course learning by stimulating students’ positive emotions (Lei et al., 2018). Furthermore, research has found that teacher support is significantly related with positive academic emotions and that students who perceive greater teacher support have greater positive emotions such as liking, enjoyment, and hope, which continue to influence their effectiveness in course learning (Sadoughi and Hejazi, 2021). During the current COVID-19 pandemic, the lack of effective teacher support and continuous guidance in online courses conducted due to the impact of the pandemic has resulted in many students having more negative academic emotions such as anxiety, depression, and despair (Nandi et al., 2021). In the current post-epidemic era, more instructor support in online learning may affect students’ learning status under the risk and normative management of the epidemic (Song et al., 2022). Therefore, in the post-epidemic context, students will feel more positive academic emotions when they have a higher level of teacher support while participating in online learning. Therefore, this study proposed the following research hypothesis:

*H2*: PTS has a positive effect on OAE.

#### 2.2.2. The effect of online academic self-efficacy on online academic emotions

During the learning process, learners’ emotions, cognition, and learning states are intertwined and contribute to their behavioral outcomes (De Carolis et al., 2021). In particular, positive emotions can influence learners’ learning performance through individual factors such as enhanced self-efficacy and self-regulation (Yu et al., 2022). Especially since the COVID-19 pandemic, some students have been prone to negative emotions such as anxiety, depression, isolation, and even the inability to consistently complete online learning tasks due to the impact of the epidemic prevention and control quarantine (García-Álvarez et al., 2021). Moreover, studies have also found that pandemic-induced disruptions and uncertainty affect students’ academic self-efficacy to some extent, which may lead to negative emotions such as anxiety, worry, and fear (Berman et al., 2022). In contrast, in online learning, more academic self-efficacy was found to be positively associated with more positive emotions (Soncini et al., 2021). That is, students who have a higher sense of academic self-efficacy in an online learning environment will feel more positive emotions such as pleasure. Therefore, in the post-epidemic era, this study proposed the following research hypotheses:

*H3*: OAS-E has a positive effect on OAE.

#### 2.2.3. Effects of online academic self-efficacy and online academic emotions on sustainable online learning engagement

Learning engagement refers to a state of focus, energy, and dedication of learners in a learning environment to consistently complete learning tasks (Schaufeli et al., 2002). Moreover, it is often used to assess student online learning outcomes and performance (Alamri, 2022). In the case of online course learning, academic self-efficacy is seen as the confidence and beliefs that learners have about taking online courses such as MOOCs and SPOCs, and is closely linked to learners’ motivation and behavior, which continues to influence their persistence in continuing to complete online learning (Adeshola and Agoyi, 2022). In addition, students’ academic self-efficacy can generate more motivation in online learning environments due to the ongoing influence of the epidemic (Kuo et al., 2021). Due to the duration of online courses and the need for more independent engagement, students’ OAS-E plays a critical role in their continued participation in online learning (Gao et al., 2021). That is, in the post-epidemic context, those students with higher OAS-E will have higher SOLE. Therefore, in the post-epidemic era, this study proposed the following research hypotheses:

*H4*: OAS-E has a positive effect on SOLE.

During the COVID-19 pandemic, the state of sustainable student engagement in learning was often considered a key indicator of learning in online courses (Adeshola and Agoyi, 2022), as the level of sustainable student engagement in online learning affects not only the behavioral performance of students but also the educational quality of online courses (Poon et al., in press). In contrast, in online learning environments, students’ academic emotions serve as an important individual factor, and their emotional state continues to influence learning interactions and engagement (Wang et al., 2021). Related research found that during the COVID-19 pandemic, students experiencing more positive and fewer negative academic emotions would have more positive interactions with teachers, peer interactions, and a greater willingness to stay engaged in online course learning (Yu et al., 2022). On the contrary, the epidemic lockdown brings great negative emotions to students, which tend to produce depression, loneliness, and anxiety. If these negative academic emotions are not eliminated in time, they will continue to affect students’ engagement and may even lead to problems such as boredom and academic suspension (Hassan et al., 2021). More importantly, due to the impact of the epidemic, unlike other global online learners, Chinese university students are prone to negative emotions such as anxiety and depression in online learning, which may be detrimental to their participation in online learning due to the stricter and more persistent embargo measures in China (Yang et al., 2021). Therefore, this study proposed the following research hypothesis:

*H5:* OAE has a positive effect on SOLE.

#### 2.2.4. Effect of sustainable online learning engagement on the academic persistence of online learning

The concept of lifelong learning has become an important issue in SDG4 (Ossiannilsson, 2022), and in the current post-epidemic era, students, as key individuals in educational sustainability, are influencing the achievement of the SDGs by their learning status and their ability to complete their education sustainably (Hanemann, 2019). Related research has also found that the behavioral, cognitive, and affective levels of student engagement in online learning in MOOC learning environments consistently influence their learning behavior outcomes and are strongly associated with the persistence of the MOOC learning environment (Jung and Lee, 2018; Kuo et al., 2021). Moreover, studies have found that students’ online learning engagement during the COVID-19 pandemic was strongly related with learning persistence, with higher online learning engagement having higher learning persistence (Adeshola and Agoyi, 2022). In other words, students will have higher OAP when they have a higher level of SOLE. Therefore, in the post-epidemic era, this study proposed the following research hypothesis:

*H6*: SOLE has a positive effect on OAP.

## 3. Materials and methods

### 3.1. Research procedure

The sample of this study was drawn from a comprehensive university in Guizhou province, China, where online courses had been offered before the beginning of the epidemic and students had experience in online learning. And the impact of COVID-19 required online teaching during the period of this study, so this study used convenience sampling to distribute online questionnaires to 593 university students in Guizhou province, China, through the Wenjuanxing platform.

Chinese university students were invited to fill out online questionnaires on social media platforms such as WeChat and QQ. The online questionnaire for this study was collected from May 16 to June 20, 2022.

### 3.2. Participants

To better investigate the relationship between teacher support and university students’ SOLE and OAP, 593 questionnaires were collected, and after deleting those questionnaires with incomplete or short response times (less than 1 min), 550 were considered valid, with an effective rate of 92.7%. Additional respondent background information is shown in Table 1.

**Table 1:** Demographic details of participants.

Variables	Content
Gender	145 males (26.4%)
	405 females (73.6%)
University	195 freshmen (35.5%)
	158 sophomores (28.7%)
	197 juniors (35.8%)
Areas of expertise	423 students (76.9%) in humanities and social sciences online courses
	127 students (23.1%) in natural science online courses
Online course platforms	126 students (22.9%) used DingTalk
	189 students (34.3%)used VooV Meeting
	46 students (8.4%) used ZOOM
	103 students (18.7%) used WeChat
	51 students (9.3%) used QQ
	35students (6.4%) used other platforms
Online learning experience before COVID-19	117 students (21.3%) had online learning experience prior to the COVID-19 pandemic
	433 students (78.7%) had no previous online learning experience

### 3.3. Measurement

This study used a quantitative research method to conduct this study. The questionnaire method is a social science research method that collects data through a cross-sectional approach and infers the characteristics of the aggregate by sampling to represent the aggregate data while controlling for errors (Kotrlik and Higgins, 2001). At the same time, the questionnaire method has the advantage of inferring the total with a small sample of data, and has the characteristics of rapidity, convenience, and reliability (Nayak and Narayan, 2019). Therefore, the measurement instrument of this study was revised and developed from previous studies and theories, and the fluency and comprehension of the questionnaire were further confirmed by inviting three experts in the field of digital learning for expert validity review and revision. We also invited 10 Chinese university students to complete the online questionnaire as part of the development process. In addition, in order to better measure the research scale, improve the validity of the completed responses and facilitate statistical analysis, this study used closed-ended questions to measure the research participants.

#### 3.3.1. Perceived teacher support

Perceived teacher support (PTS) is defined as a behavior that provides students with targeted instruction and promotes active learning in the course learning environment. It is a process that gives students more autonomy, competence, and emotional support (Liu et al., 2021). Thus, according to the above definitions, this study adapted Teachers Support Scale (TSS) of Stornes et al. (2008) with 13 questions to assess university students’ perceptions of receiving teacher support during the post-epidemic period. Example questions include “The teachers are patient in listening to our ideas and ways of doing things in the online course” and “Whenever I have problems in the online course, the teachers will provide me with timely help.”

#### 3.3.2. Online academic self-efficacy

According to definition of Bandura (1986) of self-efficacy (S-E), S-E refers more to an individual’s cognitive and psychological state of confidence and beliefs about completing specific learning tasks in the learning process (Hong et al., 2021a,b). Consequently, according to the above definition, this study adapted S-E Questionnaire (Grit-S) of Bandura (1986) to measure OAS-E with a total of 10 questions to assess the OAS-E of university students during the post-epidemic period. Examples of questions are: “When I have problems with online learning, I can usually think of some solutions” and “When I study online, no matter what problems I encounter, I believe I can figure out how to solve them.”

#### 3.3.3. Online academic emotions

Academic emotion is a learning experience that encompasses both positive and negative emotions, with positive emotions including enjoyment of learning, hope, and pride. On the other hand, negative emotions including anger, boredom, and anxiety can promote or decrease student behavior and subsequent academic achievement (Kohoulat et al., 2017). Consequently, according to the above definition, this study adapted the Achievement Emotions Questionnaire (AEQ) developed by Pekrun et al. (2011) with eight questions to assess the academic emotions of university students during the post-epidemic period. Examples of questions are: “I like studying online” and “I feel irritated when studying online.”

#### 3.3.4. Sustainable online learning engagement

Engagement in learning is widely defined as the level of energy, focus, and dedication an individual exhibits when engaged in learning activities (Wei et al., 2022). Based on these definitions, this study adapted Schaufeli et al.’s (2002) Work Engagement Questionnaire (WE), with nine questions, to assess the engagement of university students in online sustainable learning during the post-epidemic period. Example questions include: “I feel sustainably energized in online courses” and “I feel sustainably happy in online courses.”

#### 3.3.5. Online academic persistence

Online academic persistence (OAP) is a measure used to assess students’ willingness to continue their education (Adeshola and Agoyi, 2022). Based on these definitions, this study adapted Student Persistence Questionnaire (SP) of Shin (2003) with six questions to assess the OAP of university students during the post-epidemic period. Example questions are: “No matter how difficult it is, I will continue to complete the online course” and “I will continue to take online courses in the future.”

### 3.4. Data analysis

Structural equation modeling is a way to measure the structural relationships between individual study variables, to explain the hypothesized associations between individual indicators by reducing measurement error (Lee Helm et al., in press), and to assess the validity of measurement models by examining model constructs (Deng et al., 2018). Thus, in this study, after item analysis and reliability analysis using SPSS statistical software, the reliability of the study scale was confirmed through validation factor analysis. This study investigated the association between PTS, OAS-E, OAE, SOLE, and OAP by analyzing structural equation modeling tests using the AMOS 24.0 software.

## 4. Results and discussion

### 4.1. Item analysis

In order to test the fit indices of the items by identifying the original items that did not meet the criteria, this study conducted item analysis through first-order validated factor analysis (Kline, 2015). When the *χ*^2/^*df* is less than 5, the RMSEA is lower than 0.1, and the GFI is greater than 0.80, then the item has a good fit (Hair et al., 2019). Those items with factor loadings (FL) lower than 0.5 should be deleted. As a result, PTS decreased from 13 to 10 questions; OAS-E decreased from 10 to seven questions; OAE decreased from eight to seven questions; SOLE decreased from nine to eight questions; and OAP decreased from six to five questions, as seen in Table 2.

**Table 2:** First-order CFA.

Construct	*χ*2	*df*	*χ*^2^/*df*	RMSEA	GFI	AGFI	FL
Threshold	–	–	<5	<0.10	>0.80	>0.80	>0.5
PTS	73.14	17.05	4.29	0.77	0.94	0.91	0.72–0.82
OAS-E	53.43	14	3.82	0.71	0.97	0.94	0.60–0.78
OAE	56.65	14	4.05	0.74	0.97	0.94	0.64–0.76
SOLE	69.02	20	3.45	0.67	0.97	0.94	0.66–0.76
OAP	6.12	2	3.06	0.61	0.99	0.97	0.63–0.81

### 4.2. Reliability and validity analysis

In structural equation modeling, the reliability and validity of statistical data can be assessed by testing the reliability of the study constructs (Henseler et al., 2016). To measure the intrinsic consistency of a construct, a Cronbach’s alpha value with a Composite Reliability (CR) value higher than 0.7 (Hair et al., 2011) indicates a good intrinsic consistency of the construct. In the current study, Cronbach’s *α* values were from 0.79 to 0.96 and CR values ranged from 0.82 to 0.94, both of which were greater than 0.7, demonstrating good reliability of the data in this study (see Table 3).

**Table 3:** Reliability and validity analysis.

Construct	*M*	*SD*	*α*	FL	CR	AVE	*t*
PTS	3.83	0.66	0.96	0.78	0.94	0.61	18.61–21.84
OAS-E	3.72	0.54	0.91	0.72	0.89	0.53	13.01–14.05
OAE	3.34	0.71	0.91	0.71	0.88	0.51	15.57–19.33
SOLE	3.55	0.61	0.92	0.70	0.89	0.53	14.75–17.53
OAP	3.99	0.58	0.79	0.72	0.82	0.53	12.74–15.15

In addition, values of FL and Average Variance Extracted (AVE) higher than 0.5 indicate good convergent validity of the construct (Hair et al., 2011). In this study, FL values ranged from 0.72 to 0.82 for PTS, 0.60 to 0.78 for OAS-E, 0.64 to 0.76 for OAE, 0.66 to 0.76 for SOLE, and 0.63 to 0.81 for OAP. In contrast, the AVE values of the constructs in this study ranged from 0.51 to 0.61, which were above 0.5 and so had good validity.

In addition, when the value of the square root of AVE is greater than the relevant value of each construct, it is considered as having good discriminant validity (Awang, 2015). In this study, the square root of AVE for each construct was larger than the relevant value of the construct (see Table 4).

**Table 4:** Discrimination validity analysis.

Construct	1	2	3	4	5
(1) PTS	0.78				
(2) OAS-E	0.48	0.73			
(3) OAE	0.32	0.36	0.71		
(4) SOLE	0.25	0.35	0.46	0. 73	
(5) OAP	0.25	0.21	0.28	0.40	0.73

### 4.3. Model fit analysis

SEM analysis can examine the overall fit and differential acceptance of the study model (Stanley and Edwards, 2016). When the value of *χ*^2^/*df* is smaller than 5 (Hair et al., 2010); RMSEA is smaller than 0.1; GFI, AGFI, NFI, NNFI, CFI, IFI, and RFI are all higher than 0.800 (Abedi et al., 2015), and PNFI and PGFI are both larger than 0.500 (Hair et al., 2010), it indicates that the study model has good fitness. In this study, the fit values of the study model were as follows: χ^2^/*df* = 2.04, RMSEA = 0.04, GFI = 0.89, AGFI = 0.87, NFI = 0.89, NNFI = 0.94, CFI = 0.94, IFI = 0.94, RFI = 0.88, PNFI = 0.83, and PGFI = 0.78, indicating good model fitness.

### 4.4. Path analysis

**Figure 2:**
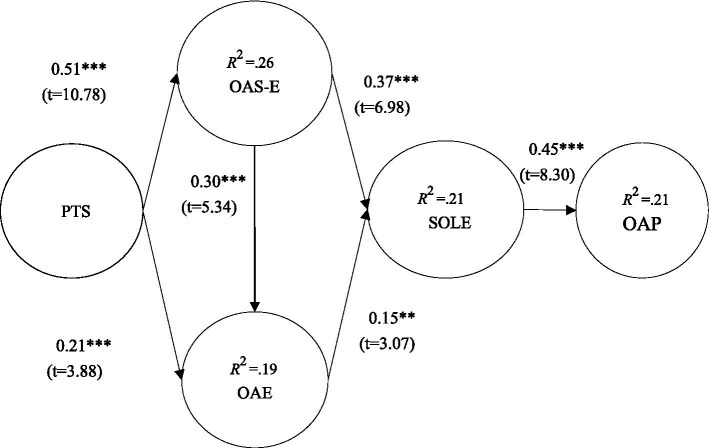
Validation of the research model. **p < 0.01, ***p < 0.001, perceived teacher support (PTS), online academic self-efficacy (OAS-E), online academic emotions (OAE), sustainable online learning engagement (SOLE), and online academic persistence (OAP).

This study proposed six research hypotheses and conducted research model validation based on learning needs resources. Results indicated that PTS had a positive influence on OAS-E (*β* = 0.51***; *t* = 10.78) and OAE (*β* = 0.21***; *t* = 3.88); OAS-E had a positive influence on OAE (*β* = 0.30***; *t* = 5.34); OAS-E (*β* = 0.37***; *t* = 6.98) and OAE (*β* = 0.15**; *t* = 3.07) had a positive influence on SOLE; and SOLE had a positive influence on OAP (*β* = 0.45***; *t* = 8.30), seen in Figure 2.

In addition, Hair et al. (2010) suggested that explanatory power values in between 0.25, 0.50, and 0.75 indicate weak, moderate, and strong levels of explanatory power. In contrast, in this study, the explanatory power of OAS-E was 26%, OAE was 19%, SOLE was 21%, and OAP was 21%; this indicates that this study had low and above moderate explanatory power, as can be seen in Figure 2.

### 4.5. Indirect effect analysis

Indirect effects analysis showed that PTS was indirectly positively related to SOLE and OAP (*β* = 0.41***, *β* = 0.39***); OAS-E was indirectly positively correlated with OAP (*β* = 0.43***), and OAE and SOLE were indirectly positively correlated with OAP (*β* = 0.13**) with 95% confidence intervals excluding 0 (**p < 0.01, ***p < 0.001), as can be seen in Table 5.

**Table 5:** Indirect effect analysis.

Construct	PTS		OAS-E		OAE	
	*β*	95% CI	*β*	95% CI	*β*	95% CI
SOLE	0.41***	[0.18, 0.32]				
OAP	0.39***	[0.08, 0.16]	0.43***	[0.13, 0.26]	0.13**	[0.02, 0.12]

** *p* < 0.01.

*** *p* < 0.001.

### 4.6. Discussion

#### 4.6.1. Perceived teacher support has a positive effect on online academic self-efficacy and academic emotion

The results of this study indicated that PTS had a positive effect on students’ OAS-E and academic emotion; that is, when students perceived higher teacher support it would be conducive to higher OAS-E and positive academic emotion, consistent with previous research. For example, Research indicates that teachers play an important role in educational sustainability (Bengtsson et al., 2020), not only by teaching the curriculum and providing more support to promote student behavior and outcomes (Lauermann and ten Hagen, 2021). Furthermore, Alamri (2022) stated that due to the COVID-19 pandemic, greater faculty support plays a crucial role in online courses such as MOOCs and SPOCs offered by schools because faculty support promotes students’ academic self-efficacy and further motivates students to stay engaged in online courses. In addition, Adeshola and Agoyi (2022) indicated that students generally had low academic self-efficacy during the COVID-19 epidemic because teachers were less likely to have positive and sustainable interactions with them. In other words, more teacher support was strongly related with higher academic self-efficacy among students and was also positively related with positive academic emotion (Lei et al., 2018). In addition, Nandi et al. (2021) indicated that students’ negative emotions were more prominent during the COVID-19 epidemic and that more teacher support was beneficial for reducing their negative emotions. As Sadoughi and Hejazi (2021) argued, teacher support is closely related to positive academic emotions such as student liking and pleasure. However, there are fewer studies on online learning in the post-epidemic era, so this study will further deepen the research on perceived teacher support, online academic self-efficacy, and academic emotions based on the post-epidemic era context.

#### 4.6.2. Online academic self-efficacy has a positive effect on academic emotion

The results of this study showed that OAS-E had a positive effect on students’ online academic mood, that is, when students perceived higher OAS-E it would be conducive to a more positive academic mood, consistent with previous research. In contrast, Yu et al. (2022) stated that when learners have good positive emotions, their academic self-efficacy is enhanced and affects both their academic behavior and performance. Meanwhile, Soncini et al. (2021) argued that students’ academic self-efficacy in online learning courses can motivate them to have positive emotions such as pleasure and liking in online courses. In addition, García-Álvarez et al. (2021) indicated that during the epidemic prevention and control period, students are prone to some negative emotions such as loneliness and anxiety in online courses if their academic self-efficacy is low. In summary, in the post-epidemic context, when students gain higher OAS-E, they are more likely to have positive emotions in online course learning. Therefore, in the process of achieving the SDGS4 goals, it is more important to stimulate students’ online academic self-efficacy and acquire positive academic emotions in order to sustain a positive learning state. However, there are fewer studies on academic self-efficacy and academic emotions of online learning among university students in the post-epidemic era. Therefore, this study will further deepen the research on online academic self-efficacy and academic emotions based on the post-epidemic era context.

#### 4.6.3. Online academic self-efficacy and online academic emotions have a positive effect on sustainable online learning engagement

The results of this study indicated that students’ OAS-E and OAE had a positive effect on SOLE; that is, when students perceived higher OAS-E, more positive academic emotions would be conducive to promoting their SOLE, which is consistent with previous studies. Adeshola and Agoyi (2022) stated that with the widespread use of online courses such as MOOCs and SPOCs during the epidemic, students’ academic self-efficacy is critical because it not only affects their behavior but also continues to affect their OAP in online learning. In addition, Gao et al. (2021) indicated that in online learning, students need to engage more autonomously, so higher perceived academic self-efficacy facilitates their sustainable engagement in online courses. That is, during the COVID-19 pandemic, students’ online self-efficacy was strongly associated with SOLE (Poon et al., in press). In contrast, Wang et al. (2021) concluded that students’ academic emotions are critical in online learning, and that higher positive emotions consistently affect students’ online engagement. Therefore, Hassan et al. (2021) stated that in the COVID-19 pandemic environment, negative academic emotions of students in online courses will affect their engagement in online or live classes and may even have negative effects such as disruptions and aversion to learning.

#### 4.6.4. Sustainable online learning engagement has a positive effect on online academic persistence

The results of this study indicated that students’ SOLE had a positive effect on OAP, consistent with previous studies. In contrast, Hanemann (2019) argued that student learning status and OAP are critical to the SDG4 agenda of the Education Sustainable Development Goals. Furthermore, Jung and Lee (2018) suggested that higher student engagement in learning is associated with higher OAP in online learning environments. Moreover, Adeshola and Agoyi (2022) more explicitly stated that during the COVID-19 pandemic, students’ behavioral, cognitive and emotional engagement in online courses would influence their sustainable and active participation in those courses. Students who have a higher commitment to online learning have more sustainable OAP (Alamri, 2022). That is, SDG4 goals are more focused on sustainable and lifelong learning for students, and in the current post-epidemic era when students perceived higher OAS-E, more positive academic emotions would be conducive to promoting their SOLE.

## 5. Conclusion and recommendations

### 5.1. Conclusion

In the development agenda of Education SDG4, there is a greater focus on lifelong learning and development of learners (Campbell et al., 2022). However, after more than 2 years of the ongoing impact of the COVID-19 epidemic, the current post-epidemic era not only affects the achievement of educational sustainability goals, but also makes student engagement in online learning an important issue in the post-epidemic era (Alamri, 2022). However, for Chinese university students, students’ OAS-E is low and their engagement in online learning is not promising (Yang et al., 2021). Also, as teachers are forced to choose online courses and adopt a non-face-to-face teaching style (Ye et al., 2022), how to provide more teacher support to promote students’ OAS-E and positive academic emotions has become an important issue for the higher education sector, as it continues to affect student engagement and OAP in online learning.

Starting from SDG4, this study used structural equation modeling to explore the relationship between university students’ PTS, SOLE, and OAP, based on the SD-R. The results of the study found that the higher the students’ PTS, the higher the OAS-E and OAE would be in the post-epidemic context. That is, in the current post-epidemic context, more PTS has a facilitating effect on students’ personal resources in the online learning environment and is more conducive to stimulating students’ self-efficacy and having positive OAE and hope in the online learning environment. Therefore, in the post-epidemic context, more instructor support in students’ online learning environment can enhance their OAS-E, which would further enhance their sustainable learning engagement and OAP in online courses.

In addition, higher OAS-E and positive OAE of college students will facilitate their online learning engagement status, because in the online learning environment, students’ personal resources can be further stimulated and have higher motivation to engage in online learning, which continues to influence the effect of students’ SOLE. More importantly, college students who have higher SOLE have higher OAP. In other words, when teachers conduct online courses or live classes, they should provide more resources to support students’ engagement with more vivid and imaginative cases, and guide students to participate actively and positively in online learning, thus consistently influencing their online learning engagement and OAP.

### 5.2. Recommendations

Previous studies have more often investigated the associations between online learning environments and learner behavior (Adeshola and Agoyi, 2022). However, with the advent of the post-epidemic era, concern for online learning engagement and OAP among university students has become more frequently discussed. It is more convenient and timelier for teachers to conduct online courses or live classes in the post-epidemic era, so providing more teacher support when taking online courses is not only conducive to promoting students’ OAS-E, but also inspires students to actively and continuously participate in online learning by achieving two-way interaction between teachers and students in online courses, and further maintaining OAP to achieve the goal of education sustainability.

At the same time, this study found that students’ positive academic sentiment regarding learning in online courses was low, making it particularly important to provide more instructor support resources and to reform online teaching methods. Particularly in the current post-epidemic era, the lack of face-to-face teaching opportunities and effective two-way interactions makes teacher support approaches which enhance positive student emotions particularly important when teachers are unable to effectively identify students’ emotions while participating in online learning courses. Therefore, it is suggested that university faculty should improve their teaching strategies for online courses, enhance their skills and abilities to conduct online courses, create a relaxed and enjoyable online learning atmosphere, and diversify their online course delivery methods to stimulate students’ online positive academic emotions and further promote their SOLE and OAP.

### 5.3. Limitations and future study

This is a cross-sectional study, with data collected from May 16 to June 20, 2022. In the current post-epidemic era, the provision of additional support resources to promote student performance and persistence in online courses has become an important part of the education sustainability agenda. Therefore, the association among teacher support and students’ online learning performance and OAP after the end of COVID-19 may be explored through a longitudinal study in a follow-up study.

In addition, more consideration was given to the behavioral and attitudinal aspects of the study participants. However, it was not possible to fully interpret the phenomena and the underlying deeper reasons behind the findings of this study. Thus, in-depth interviews could be used in follow-up studies to further explore university students’ views of PTs versus their SOLE and academic emotions.

In addition, research has found that learning resources in the learning environment can enhance student engagement and motivate students to be consistently and actively engaged in their learning activities. Therefore, in follow-up studies, other types of learning resources such as social support, family support, or peer support may be explored to further extend this study’s findings on the impact of students’ online learning performance and OAP.

In addition, the participants of this study are from a university in Guizhou Province, China, and since there are more female students than male students in this university, and the participants of this study are predominantly female, the scope of the participants will be expanded in future studies to further expand the gender ratio of the subjects in the context of China.

## Data availability statement

The original contributions presented in the study are included in the article/supplementary material, further inquiries can be directed to the corresponding author.

## Ethics statement

Ethical review and approval was not required for the study on Human Participants in accordance with the Local Legislation and Institutional Requirements. Written informed consent from the participants was not required to participate in this study in accordance with the National Legislation and the Institutional Requirements.

## Author contributions

All authors listed have made a substantial, direct, and intellectual contribution to the work and approved it for publication.

## Conflict of interest

The authors declare that the research was conducted in the absence of any commercial or financial relationships that could be construed as a potential conflict of interest.

## Publisher’s note

All claims expressed in this article are solely those of the authors and do not necessarily represent those of their affiliated organizations, or those of the publisher, the editors and the reviewers. Any product that may be evaluated in this article, or claim that may be made by its manufacturer, is not guaranteed or endorsed by the publisher.

## References

[ref01] Abedi G. Rostami F. Nadi A. (2015). Analyzing the dimensions of the quality of life in hepatitis B patientsusing confirmatory factor analysis. Glob. J. Health Sci. 7, 22–31. doi: 10.5539/gjhs.v7n7p22 26153200 PMC4803937

[ref1] Adeshola I. Agoyi M. (2022). Examining factors influencing E-learning engagement among university students during Covid-19 pandemic: a mediating role of “learning persistence”. Interact. Learn. Environ., 1–28. doi: 10.1080/10494820.2022.2029493

[ref2] Alamri M. M. (2022). Investigating students’ adoption of MOOCs during COVID-19 pandemic: students’ academic self-efficacy, learning engagement, and learning persistence. Sustainability 14:714. doi: 10.3390/su14020714

[ref02] Awang Z. (2015). Sem made simple, a gentle approach to learning structural equation modeling. Kajang: MPWS Rich.

[ref3] Bandura A. (1977). Self-efficacy: toward a unifying theory of behavioral change. Psychol. Rev. 84, 191–215. doi: 10.1037/0033-295X.84.2.191, PMID: 847061

[ref4] Bandura A. (1986). The explanatory and predictive scope of self-efficacy theory. J. Soc. Clin. Psychol. 4, 359–373. doi: 10.1521/jscp.1986.4.3.359

[ref5] Bengtsson S. Kamanda M. Ailwood J. Barakat B. (2020). “Teachers are more than ‘supply’: toward meaningful measurement of pedagogy and teachers in SDG 4” in Grading Goal Four. ed. Wulff A. (Boston: Brill), 214–237.

[ref6] Berga K. A. Vadnais E. Nelson J. Johnston S. Buro K. Hu R.. (2021). Blended learning versus face-to-face learning in an undergraduate nursing health assessment course: a quasi-experimental study. Nurse Educ. Today 96:104622. doi: 10.1016/j.nedt.2020.104622, PMID: 33125980

[ref7] Berman A. H. Bendtsen M. Molander O. Lindfors P. Lindner P. Granlund L.. (2022). Compliance with recommendations limiting COVID-19 contagion among university students in Sweden: associations with self-reported symptoms, mental health and academic self-efficacy. Scand. J. Public Health 50, 70–84. doi: 10.1177/1403494821102782, PMID: 34213359 PMC8808007

[ref8] Campbell C. Hobbs L. Xu L. McKinnon J. Speldewinde C. (2022). Girls in STEM: addressing SDG 4 in context. Sustainability 14:4897. doi: 10.3390/su14094897

[ref9] Cheng Y. Liu H. Wang S. Cui X. Li Q. (2021). Global action on SDGs: policy review and outlook in a post-pandemic era. Sustainability 13:6461. doi: 10.3390/su13116461

[ref10] De Carolis B. D’Errico F. Macchiarulo N. Paciello M. Palestra G. (2021). “Recognizing cognitive emotions in E-learning environment,” in Bridges and Mediation in Higher Distance Education. HELMeTO 2020. Communications in Computer and Information Science, vol 1344. Cham: Springer.

[ref11] Deng L. Yang M. Marcoulides K. M. (2018). Structural equation modeling with many variables: a systematic review of issues and developments. Front. Psychol. 9:580. doi: 10.3389/fpsyg.2018.00580, PMID: 29755388 PMC5932371

[ref12] Fagö-Olsen H. Lynggaard C. D. Aanæs K. Cayé-Thomasen P. Andersen S. A. W. (2020). Developing a national e-learning course in otorhinolaryngology: the Danish experience. Eur. Arch. Otorhinolaryngol. 277, 1829–1836. doi: 10.1007/s00405-020-05889-w, PMID: 32170422

[ref14] Gao H. Ou Y. Zhang Z. Ni M. Zhou X. Liao L. (2021). The relationship between family support and E-learning engagement in college students: the mediating role of e-learning normative consciousness and behaviors and self-efficacy. Front. Psychol. 12:573779. doi: 10.3389/fpsyg.2021.573779, PMID: 33613373 PMC7890012

[ref15] García-Álvarez D. Hernández-Lalinde J. Cobo-Rendón R. (2021). Emotional intelligence and academic self-efficacy in relation to the psychological well-being of university students during COVID-19 in Venezuela. Front. Psychol. 12:759701. doi: 10.3389/fpsyg.2021.759701, PMID: 34975650 PMC8715985

[ref16] Hair J. F. Jr. Black W. C. Babin B. J. Anderson R. E. (2010). Anderson Multivariate Data Analysis. 7th Edn. Upper Saddle River: Prentice Hall.

[ref06] Hair J. P. Black J. P. Babin J. P. Anderson R. E. (2019). Multivariate Data Analysis, 8th Edn. Harlow: Cengage Learning.

[ref17] Hair J. F. Ringle C. M. Sarstedt M. (2011). PLS-SEM: indeed a silver bullet. J. Market. Theory Pract. 19, 139–152. doi: 10.2753/MTP1069-6679190202

[ref18] Hanemann U. (2019). Examining the application of the lifelong learning principle to the literacy target in the fourth sustainable development goal (SDG 4). Int. Rev. Educ. 65, 251–275. doi: 10.1007/s11159-019-09771-8

[ref19] Hassan S. U. N. Algahtani F. D. Atteya M. R. Almishaal A. A. Ahmed A. A. Obeidat S. T.. (2021). The impact of extended E-learning on emotional well-being of students during the COVID-19 pandemic in Saudi Arabia. Children 9:13. doi: 10.3390/children9010013, PMID: 35053638 PMC8774542

[ref20] Henseler J. Hubona G. Ray P. A. (2016). Using PLS path modeling in new technology research: updated guidelines. Ind. Manag. Data Syst. 116, 2–20. doi: 10.1108/IMDS-09-2015-0382

[ref21] Hong J. C. Lee Y. F. Ye J. H. (2021a). Procrastination predicts online self-regulated learning and online learning ineffectiveness during the coronavirus lockdown. Personal. Individ. Differ. 174:110673. doi: 10.1016/j.paid.2021, PMID: 33551531 PMC7846229

[ref22] Hong J. C. Zhang H. L. Ye J. H. Ye J. N. (2021b). The effects of academic self-efficacy on vocational students behavioral engagement at school and at firm internships: a model of engagement-value of achievement motivation. Educ. Sci. 11:387. doi: 10.3390/educsci11080387

[ref23] Jdaitawi M. (2020). Does flipped learning promote positive emotions in science education? A comparison between traditional and flipped classroom approaches. Elect. J. E-Learn. 18, 516–524. doi: 10.34190/JEL.18.6.004

[ref24] Jin Y. Q. Lin C. L. Zhao Q. Yu S. W. Su Y. S. (2021). A study on traditional teaching method transferring to E-learning under the COVID-19 pandemic: from Chinese students' perspectives. Front. Psychol. 12:632787. doi: 10.3389/fpsyg.2021.632787, PMID: 33776854 PMC7991594

[ref25] Jung Y. Lee J. (2018). Learning engagement and persistence in massive open online courses (MOOCS). Comput. Educ. 122, 9–22. doi: 10.1016/j.compedu.2018.02.013

[ref26] Kim H. J. Hong A. J. Song H. D. (2019). The roles of academic engagement and digital readiness in students’ achievements in university e-learning environments. Int. J. Educ. Technol. High. Educ. 16:21. doi: 10.1186/s41239-019-0152-3

[ref27] Kline R. B. (2015). Principles and Practice of Structural Equation Modeling. 4th Edn. New York: Guilford Publications.

[ref28] Kohoulat N. Hayat A. A. Dehghani M. R. Kojuri J. Amini M. (2017). Medical students’ academic emotions: the role of perceived learning environment. J. Adv. Med. Educ. Prof. 5, 78–83. PMID: 28367464 PMC5346172

[ref29] Koob C. Schröpfer K. Coenen M. Kus S. Schmidt N. (2021). Factors influencing study engagement during the COVID-19 pandemic: a cross-sectional study among health and social professions students. PLoS One 16:e0255191. doi: 10.1371/journal.pone.0255191, PMID: 34314450 PMC8315536

[ref30] Kotrlik J. W. K. J. W. Higgins C. C. H. C. C. (2001). Organizational research: determining appropriate sample size in survey research appropriate sample size in survey research. Inf. Technol. Learn. Perform. J. 19:43

[ref31] Kuo T. M. Tsai C. C. Wang J. C. (2021). Linking web-based learning self-efficacy and learning engagement in MOOCs: the role of online academic hardiness. Internet High. Educ. 51:100819. doi: 10.1016/j.iheduc.2021.100819

[ref32] Lauermann F. ten Hagen I. (2021). Do teachers’ perceived teaching competence and self-efficacy affect students’ academic outcomes? A closer look at student-reported classroom processes and outcomes. Educ. Psychol. 56, 265–282. doi: 10.1080/00461520.2021.1991355

[ref33] Lee Helm J. Langenberg B. Grossman E. Poulin J. Mayer A. (in press). Using structural equation modeling in place of between-subjects analysis of variance. Struct. Equ. Model. 30, 123–131. doi: 10.1080/10705511.2022.2033977

[ref34] Lei H. Cui Y. Chiu M. M. (2018). The relationship between teacher support and students' academic emotions: a meta-analysis. Front. Psychol. 8:2288. doi: 10.3389/fpsyg.2017.02288, PMID: 29403405 PMC5786576

[ref04] Lee J. Song H. D. Hong A. J. (2019). Exploring factors, and indicators for measuring students’ sustainable engagement in e-learning. Sustainability 11:985. doi: 10.3390/su11040985

[ref35] Lesener T. Pleiss L. S. Gusy B. Wolter C. (2020). The study demands-resources framework: an empirical introduction. Int. J. Environ. Res. Public Health 17:5183. doi: 10.3390/ijerph17145183, PMID: 32709128 PMC7400357

[ref36] Liu X. X. Gong S. Y. Zhang H. P. Yu Q. L. Zhou Z. J. (2021). Perceived teacher support and creative self-efficacy: the mediating roles of autonomous motivation and achievement emotions in Chinese junior high school students. Think. Skills Creat. 39:100752. doi: 10.1016/j.tsc.2020.100752

[ref37] Liu Z. Yang C. Rüdian S. Liu S. Zhao L. Wang T. (2019). Temporal emotion-aspect modeling for discovering what students are concerned about in online course forums. Interact. Learn. Environ. 27, 598–627. doi: 10.1080/10494820.2019.1610449

[ref38] Maatuk A. M. Elberkawi E. K. Aljawarneh S. Rashaideh H. Alharbi H. (2022). The COVID-19 pandemic and E-learning: challenges and opportunities from the perspective of students and instructors. J. Comput. High. Educ. 34, 21–38. doi: 10.1007/s12528-021-09274-2, PMID: 33967563 PMC8091987

[ref39] Nandi A. Xhafa F. Subirats L. Fort S. (2021). Real-time emotion classification using eeg data stream in e-learning contexts. Sensors 21:1589. doi: 10.3390/s21051589, PMID: 33668757 PMC7956809

[ref40] Nayak M. S. D. P. Narayan K. A. (2019). Strengths and weaknesses of online surveys. IOSR J. Humanit. Soc. Sci. 6, 31–38. doi: 10.9790/0837-2405053138

[ref41] Ossiannilsson E. S. (2022). Resilient agile education for lifelong learning post-pandemic to meet the United Nations sustainability goals. Sustainability 14:10376. doi: 10.3390/su141610376

[ref42] Pekrun R. Goetz T. Frenzel A. C. Barchfeld P. Perry R. P. (2011). Measuring emotions in students’ learning and performance: the achievement emotions questionnaire (AEQ). Contemp. Educ. Psychol. 36, 36–48. doi: 10.1016/j.cedpsych.2010.10.002

[ref43] Poon W. C. Kunchamboo V. Koay K. Y. (in press). E-learning engagement and effectiveness during the COVID-19 pandemic: the interaction model. Int. J. Hum. Comput. Interact. 1–15. doi: 10.1080/10447318.2022.2119659

[ref44] Putra R. B. Ridwan M. Mulyani S. R. Ekajaya D. S. Putra R. A. (2019). “Impact of learning motivation, cognitive and self-efficacy in improving learning quality e-learning in industrial era 4.0.” in *Paper Presented at the Journal Of Physics: Conference Series: International Conference Computer Science and Engineering (IC2SE)*, 26–27 April 2019, Vol. 1339, Padang.

[ref45] Robins T. G. Roberts R. M. Sarris A. (2015). Burnout and engagement in health profession students: the relationships between study demands, study resources and personal resources. Australas. J. Organ. Psychol. 8:e1. doi: 10.1017/orp.2014.7

[ref46] Sadoughi M. Hejazi S. Y. (2021). Teacher support and academic engagement among EFL learners: the role of positive academic emotions. Stud. Educ. Eval. 70:101060. doi: 10.1016/j.stueduc.2021.101060

[ref47] Schaufeli W. B. Martinez I. M. Pinto A. M. Salanova M. Bakker A. B. (2002). Burnout and engagement in university students: a cross-national study. J. Cross-Cult. Psychol. 33, 464–481. doi: 10.1177/0022022102033005003

[ref48] Shen G. R. An Y. (2022). Influencing factors of psychological stress under the mixed teaching mode based on SPOC+PBL. Front. Psychol. 13:979206. doi: 10.3389/fpsyg.2022.979206, PMID: 36148096 PMC9486209

[ref49] Shin N. (2003). Transactional presence as a critical predictor of success in distance learning. Distance Educ. 24, 69–86. doi: 10.1080/01587910303048

[ref50] Soncini A. Politi E. Matteucci M. C. (2021). Teachers navigating distance learning during COVID-19 without feeling emotionally exhausted: the protective role of self-efficacy. Sch. Psychol. Forum 36, 494–503. doi: 10.1037/spq0000469, PMID: 34766813

[ref51] Song W. Wang Z. Zhang R. (2022). Classroom digital teaching and college students’ academic burnout in the post COVID-19 era: a cross-sectional study. Int. J. Environ. Res. Public Health 19:13403. doi: 10.3390/ijerph192013403, PMID: 36293983 PMC9603840

[ref07] Stanley L. M. Edwards M. C. (2016). Reliability and model fit. Educ. Psychol. Meas. 76, 976–985. doi: 10.1177/0013164416638900 29795896 PMC5965612

[ref52] Stornes T. Bru E. Idsoe T. (2008). Classroom social structure and motivational climates: on the influence of teachers' involvement, teachers' autonomy support and regulation in relation to motivational climates in school classrooms. Scand. J. Educ. Res. 52, 315–329. doi: 10.1080/00313830802025124

[ref53] Tran T. Hoang A. D. Nguyen Y. C. Nguyen L. C. Ta N. T. Pham Q. H.. (2020). Toward sustainable learning during school suspension: socioeconomic, occupational aspirations, and learning behavior of vietnamese students during COVID-19. Sustainability 12:4195. doi: 10.3390/su12104195

[ref54] UN (2020). The sustainable development goals report. Available at: https://unstats.un.org/sdgs/report/2020/ (Accessed September 06, 2022).

[ref55] United Nations (2015). Sustainable Development Goals. Available at: http://www.un.org/sustainabledevelopment/sustainble-development-goals/ (Accessed September 01, 2022).

[ref56] Wang Z. Qadir A. Asmat A. Mian M. S. A. Luo X. (2021). The advent of coronavirus disease 2019 and the impact of mobile learning on student learning performance: the mediating role of student learning behavior. Front. Psychol. 12:796298. doi: 10.3389/fpsyg.2021.796298, PMID: 35211054 PMC8862787

[ref57] Wei C. Ma Y. Ye J. H. Nong L. (2022). First-year college students' mental health in the post-COVID-19 era in Guangxi, China: a study demands-resources model perspective. Front. Public Health 10:906788. doi: 10.3389/fpubh.2022.906788, PMID: 35769778 PMC9234168

[ref58] Yang J. Peng M. Y. P. Wong S. Chong W. (2021). How E-learning environmental stimuli influence determinates of learning engagement in the context of COVID-19? SOR model perspective. Front. Psychol. 12:584976. doi: 10.3389/fpsyg.2021.584976, PMID: 33868072 PMC8044515

[ref59] Ye J. H. Lee Y. S. He Z. (2022). The relationship among expectancy belief, course satisfaction, learning effectiveness, and continuance intention in online courses of vocational-technical teachers college students. Front. Psychol. 13:904319. doi: 10.3389/fpsyg.2022.904319, PMID: 35800948 PMC9253592

[ref60] Yekefallah L. Namdar P. Panahi R. Dehghankar L. (2021). Factors related to students' satisfaction with holding e-learning during the COVID-19 pandemic based on the dimensions of e-learning. Heliyon 7:e07628. doi: 10.1016/j.heliyon.2021.e07628, PMID: 34381894 PMC8334372

[ref61] Yu J. Huang C. He T. Wang X. Zhang L. (2022). Investigating students’ emotional self-efficacy profiles and their relations to self-regulation, motivation, and academic performance in online learning contexts: a person-centered approach. Educ. Inf. Technol. 27, 11715–11740. doi: 10.1007/s10639-022-11099-0

